# Symptomatic Acetabular Erosion After Hip Hemiarthroplasty: Is It a Major Concern? A Retrospective Analysis of 2477 Hemiarthroplasty Cases

**DOI:** 10.3390/jcm13226756

**Published:** 2024-11-10

**Authors:** Ahmed Nageeb Mahmoud, Michael Suk, Daniel S. Horwitz

**Affiliations:** 1 Geisinger Musculoskeletal Institute, Danville, PA 17822, USA; ahmednageeb2011@gmail.com (A.N.M.); msuk@geisinger.edu (M.S.); 2 Faculty of Medicine, Ain Shams University, Cairo 11591, Egypt

**Keywords:** hip fracture, hip hemiarthroplasty, acetabular erosion, acetabular wear

## Abstract

**Background/Objectives**: Acetabular erosion is a postoperative condition that can occur after hip hemiarthroplasty (HA), potentially leading to pain and requiring conversion to total hip arthroplasty (THA). Given the discrepancy in its incidence and impact in the literature, this study aims to report the incidence of symptomatic acetabular erosion and the subsequent conversion to THA in all HA cases performed in a single health system. **Methods**: A total of 2477 HA cases had their clinical notes and serial radiographs examined for this retrospective study. Outcome measures included any records of hip or groin pain and conversion to THA that were attributed to acetabular erosion as documented in the clinical notes. **Results**: Two thousand four hundred and seventy-seven HA cases were reviewed in this study. The mean age for all patients in the study was 81.5 years and the mean follow up was 3.7 years. Out of the 2477 HA cases, only 12 HA cases (0.48%) in 12 patients had data records of chronic hip pain, attributable to acetabular wear in the clinical notes, of variable severity and presentations. The mean duration until the clinical documentation of acetabular wear-induced pain was 25.6 months (range, 1.4–146.4 months), with most symptomatic hip erosion cases presented within the first year (50% presented within the first 6 months) after the index HA surgery. Despite that, only five cases underwent conversion to THA (0.2%) while seven patients received conservative management. **Conclusions**: In patients older than 65 years of age who are candidates for HA, the incidence of symptomatic hip erosion and the subsequent conversion to THA is low and hence HA remains a viable treatment option. Based on the duration until clinical presentation of acetabular erosion, this study suggests that the state of acetabular cartilage during surgery may influence the development of early acetabular wear in most symptomatic patients. On the other hand, hip hemiarthroplasty is a rare treatment option for displaced femoral neck fractures in patients younger than 65 years of age, and its use in this patient subset depends on conditional and patient-related factors such as the activity level, cognitive function, and medical comorbidities.

## 1. Introduction

Hip hemiarthroplasty (HA) is a widely utilized treatment option for displaced femoral neck fractures in elderly patients [1,2,3,4]. Despite the comparable incidence of major complications between HA and THA, namely dislocation, infection, and periprosthetic fractures in the literature [5,6,7,8,9,10], there is increasing evidence that supports the use of total hip arthroplasty (THA) in active elderly patients with displaced femoral neck fractures who are medically fit, have outdoor unassisted activity levels, and have no cognitive impairment, to achieve better functional outcomes and patient’s satisfaction [8,9,10,11,12,13].

Another less-reported reason that supports the THA option is the potential occurrence of acetabular wear/erosion after HA [14,15,16,17]. The incidence of acetabular wear, pain, and subsequent revision to THA varies in the literature. Radiological acetabular wear was reported to occur in 1.96–66% of HA cases, and the conversion rate to THA was reported to occur in 0.6–16.7% [14,15,16,17]. These variations in the incidences of radiographic and symptomatic acetabular erosion and subsequent conversion to THA in the literature could be attributed to the variations in the patients’ age, follow-up durations, number of studied cases, and surgeon’s interpretation of radiographic and clinical signs. The occurrence of acetabular erosion after HA was attributed to several factors, including patients’ biological age, activity level, health condition, and likely remaining life span [13,18,19]. Other identified surgical factors included limb length discrepancy [18,20], smaller HA femoral head [16,20], and lower T score [20].

With this wide variation in the incidence of acetabular wear-induced hip pain and conversion to THA in the literature, and with the relatively small number of the studied populations in the relevant literature that particularly focused on acetabular erosion, the purpose of this study is to specifically examine the incidence of symptomatic acetabular wear/erosion and subsequent conversion to THA in a larger study population. We particularly focused on symptomatic rather than radiographic acetabular wear, since it is the main factor that determines the decision behind conversion to THA in HA patients who present with acetabular wear.

## 2. Materials and Methods

Institutional board review approval was obtained to review our electronic medical records for all cases of hemiarthroplasty that were performed at our multi-hospital health system. Using the procedural codes for surgery, a total of 2477 cases of hemiarthroplasty in 2344 patients were extracted from our electronic medical records and individually reviewed. Patients’ demographics, clinical inpatient and outpatient data, operative notes, and serial radiographs were retrospectively examined against our selection criteria. The serial radiographs of all patients were examined to document and prove the presence of acetabular erosion based on Baker’s criteria [14]. Acetabular erosion has been concluded radiographically when either loss of the joint space, acetabular bone loss, bone erosion, prosthetic femoral head migration, or protrusio acetabuli [14] were noticed in the serial postoperative plain radiographs.

### 2.1. Inclusion Criteria

Any patient who underwent hip hemiarthroplasty, whether unipolar or bipolar, and who

1. has been diagnosed with acetabular erosion in the clinical notes, serial radiographs or radiology reports, and
2. presented with chronic hip, groin, or gluteal pain after HA surgery with an intensity of ≥4 on a 0–10 pain scoring chart (visual analog scale) which increases with weight-bearing, active, or passive hip movement that was attributed to acetabular erosion, as decided by the treating physician, and/or
3. underwent or was planned for revision hip arthroplasty for chronic hip or groin pain secondary to acetabular erosion as decided by the treating physician. Conversion to THA was decided by the treating surgeon based mainly on the presence of symptomatic acetabular wear that could not be controlled conservatively.

### 2.2. Exclusion Criteria

1. Patients with a history of postoperative periprosthetic infection.
2. Patients who had a positive bacterial culture that was obtained by hip aspiration, sonication, or biopsy.
3. Patients with any records of prosthetic dislocation.
4. Patients who sustained ipsilateral periprosthetic or acetabular fracture.

Hemiarthroplasty was performed at the discretion of the surgeon based on the diagnosis, patient age, and medical history. As our health system includes a level 1, tertiary referral center plus 9 other hospitals, and serves 45 counties including mostly rural areas, almost all our patients continue to receive their medical care within our healthcare system and have a continuous track of documented medical records. In this study, we depended on the available records in our electronic medical database, which also document any clinical visit or surgery that might be performed outside our health system.

Surgery was performed by 229 different surgeons using 38 different implant systems. At our institution, HA is indicated, based on the surgeon’s discretion, for patients with femoral neck displaced fractures or metastasis who have indoor or assisted outdoor activities, patients without severe hip osteoarthritis (Tönnis grades 2 or less), and patients whose fractures or medical characteristics precluded a successful fracture repair surgery, or a more complex THA surgery as decided by the surgeon. As for the patient’s age, the majority of patients (93%) who received HA in this study were 65 or older, and 62.7% were older than 85 (Table 1). For younger patients, HA is indicated for patients whose medical comorbidities, weight-bearing status, or fracture characteristics precluded receiving a THA or ORIF. To comprise all the relevant cases, we have included all the hemiarthroplasty cases whether the indication was the management of acute recent trauma, neglected trauma, pathological lesions or a revision of failed internal fixation surgery.

## 3. Statistical Analysis

Statistical analysis was performed using SPSS software, version 25 (SPSS Inc., PASW Statistics for Windows version 25. Chicago, IL, USA: SPSS Inc.). The mean values, standard deviation, and proportions were calculated. The Chi-square test was utilized to perform the subgroup analyses. A *p*-value above 0.05 is considered statistically insignificant.

## 4. Results

Two thousand, four hundred seventy-seven hemiarthroplasties in 2344 patients performed between 1988 and July 2024 were retrieved from our database. Patients comprised a total of 1568 females and 776 males (range 2.02:1) with a mean age of 81.5 years (range, 26.1 (a patient with Down syndrome) to 110 years) at the time of surgery (Table 1). Bipolar HA was used in 1368 cases while unipolar was used in 1109 cases. Cemented stems were utilized in 1793 cases, and uncemented stems were used in 684 cases. In 1559 cases, collared stems were used while uncollared stems were used in 918 cases. Significant medical comorbidities were present in 1959 cases at the time of surgery, and the most prevalent comorbidities were diabetes, chronic kidney disease, heart failure, hypothyroidism, dementia, and Parkinson’s disease. Twenty-four cases (23 patients) had pathological fractures or lesions due to either a primary bone tumor or a metastatic tumor secondary to a breast, prostate, kidney, liver, GIT, thyroid, lung, or blood malignancy.

A total of 1198 patients (51.1% of patients, 1225 cases) died at a mean of 2.3 years postoperatively, including 326 patients (327 cases) who died in the first three months after surgery (13.9% of all patients, Table 2). The mean age of the 1198 patients at the time of surgery was 82.6 years. For the remaining patients who were still alive at the time (1146 patients, 1252 cases), the mean age at the time of surgery was 80.4 years and the average follow-up was 5.03 years. The average follow-up for the entire study cohort is 3.7 years (Table 2 and Table 3).

### Symptomatic Acetabular Erosion

**Figure 1:**
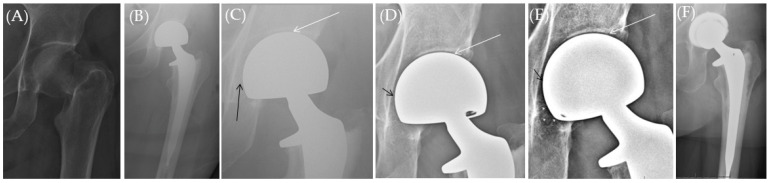
(**A**) Plain hip X-ray of a 64.7-year-old female (Case 6, Table 4) who sustained a left femoral neck fracture. (**B**,**C**) 1-day postoperative plain hip radiograph showing bipolar HA with preserved joint space (white arrow) and preserved medial acetabular bone (black arrow). (**D**) 6-week, and (**E**) 18-week follow-up plain radiograph showing stable HA with progressive reduction of the joint space (white arrow), loss of medial acetabular wall (black arrow), and focal bony loss at the acetabular teardrop (asterisks*). The patient eventually received a total hip arthroplasty (**F**) due to chronic debilitating groin pain.

**Figure 2:**
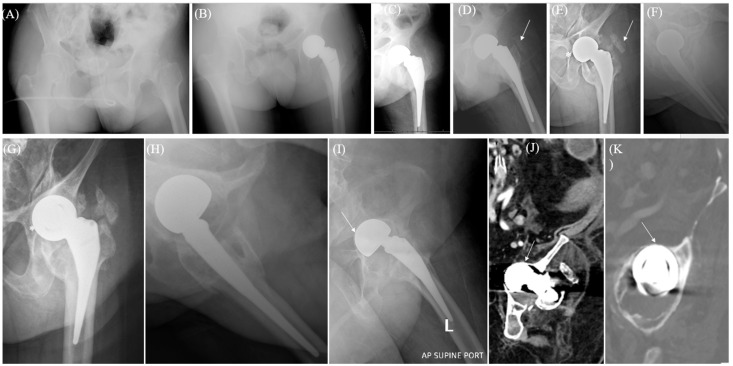
(**A**) Plain hip radiographs of a 47.1-year-old man with spastic cerebral palsy and limited indoor activities (Case 9, Table 4) who presented with a displaced left femoral neck fracture. (**B**) Immediate postoperative plain hip radiographs after cementless bipolar hemiarthroplasty. (**C**,**D**) Six-week postoperative plain pelvic radiographs showing stable components and early heterotopic ossification (HO) (arrow). (**E**,**F**) Here, 24-month postoperative plain hip radiographs showing HO (arrow) and prosthetic head protrusion through the ilioischial line (asterisk*). (**G**,**H**) A 62-month plain hip radiograph showing grade 3 HO and prosthetic head protrusion (asterisk*). At this point, the patient presented with marked groin pain (**I**–**K**) 177-month postoperative radiographs (**I**) and CT scan cuts (coronal (**J**) and sagittal (**K**)) showing an almost completely fused hip with prosthetic head intrapelvic protrusion (arrows) and class 4 HO.

**Figure 3:**
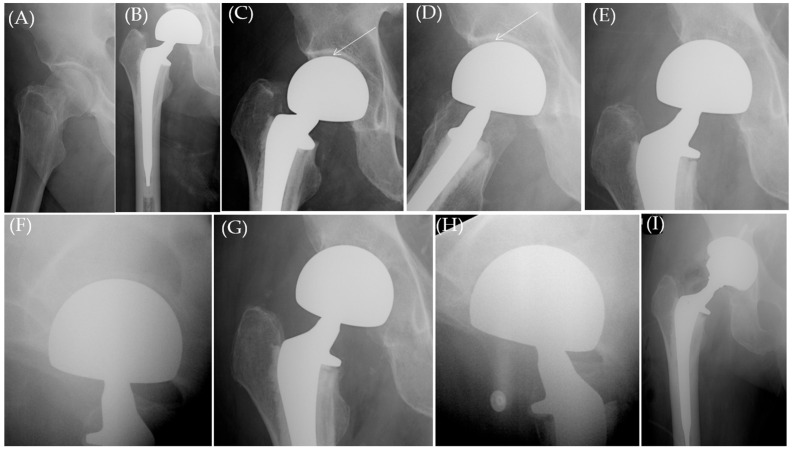
(**A**) Plain hip X-ray of a 76.5-year-old male (Case 10, Table 4) who sustained a right femoral neck fracture. (**B**–**D**) A 2-week postoperative plain hip radiograph showing bipolar HA with preserved joint space (white arrow), and (**E**,**F**) 11-week and (**G**,**H**) 22-week postoperative X-rays showing progressive loss of the joint space and advancement of the femoral head inside the acetabulum in the lateral view in comparison to the 2-week postoperative lateral radiograph (**D**). (**I**) Total hip arthroplasty performed 6 weeks later (28 weeks after the index HA surgery for debilitating groin pain).

A total of 12 patients (12 cases) had data records of chronic hip pain, attributable to acetabular wear (Figure 1, Figure 2 and Figure 3) in the clinical notes (0.48% of HA cases, Table 4), of variable severity and presentations after a mean of 25.6 months postoperative (range, 1.5–146 months). Despite that, only five cases underwent conversion to THA (0.2%) while seven received conservative management.

Evaluating the time until the presentation of significant hip pain, seven patients presented within the first year after surgery (six within the first 6 months), and another three presented within 1–3 years. Only two cases presented with chronic pain after 5 years. Seven cases had bipolar components, and five cases had unipolar components, while ten cases had collared and two had uncollared stems. Three patients were younger than 65, five were 65–80, and four were older than 80. The mean femoral head size in all twelve patients was 48 (range 40–54), and only one patient had a recorded limb lengthening of 1 cm postoperatively. No significant difference was found between the patients’ age groups nor the implant types (unipolar versus bipolar) and the incidence of symptomatic acetabular erosion in this study (Table 1 and Table 4).

Eleven other cases (eleven patients) had mild, radiographic Baker grade 1 [14] acetabular erosion that was completely asymptomatic and discovered only on the serial radiographs without being reported in the patients’ clinical records.

## 5. Discussion

This study aimed to retrospectively evaluate the incidence of symptomatic acetabular wear and conversion to THA in all hip arthroplasty cases in our multi-hospital health system. Out of 2477 hemiarthroplasty cases available for review, only 12 cases had symptomatic, radiographically evident acetabular wear that did not occur after infection, instability, or fracture, and only 5 cases underwent revision for acetabular erosion.

The small incidence of symptomatic erosion and subsequent conversion in this study could be attributed to several factors. First, the age of the included patients represents a major factor. In this study, 93% of cases were above 65 at the time of surgery, and two-thirds of these were above 80. The activity level, health condition, and remaining life span have potentially played a significant role in the reported incidence of acetabular wear and related pain. Additionally, more than one-third of the study population (35.7% of cases) died within 3 years of surgery. Although this mortality rate aligns with the reported mortality rates following hip fractures in the literature (1-year mortality reaching 33% and midterm mortality reaching 79% in patients older than 65 years [22,23]), the time available for the patients to develop acetabular wear throughout the study was obviously reduced. Similarly, the potential tendency towards conservative treatment of symptomatic acetabular wear by the treating physician in patients in this age group must have influenced this reported low incidence of conversion to THA. Other factors including comorbidities considered too severe for revision surgery, psychosocial, and financial factors may have played a key role in the reported conversion rate.

Acetabular wear following hemiarthroplasty is a concern that is usually raised when hemiarthroplasty is offered to treat a hip fracture. The worry about its occurrence may drive the decision towards performing total joint replacement, a longer and more expensive surgery, to achieve higher reported patient satisfaction and a decrease in the incidence of revision due to pain [24]. Another consideration may also be the increased morbidity, mortality, and cost of revision hip arthroplasty (in case of acetabular erosion) compared to primary arthroplasty [25]. Total hip replacement itself, is not without complications, however, as it has an overall incidence of revision and mortality that is almost comparable to hemiarthroplasty [9,26]. Considering the available outcomes in the literature, patients’ age and life expectancy, and the results of this study, surgeons may confidently offer hemiarthroplasty in patients older than 80 years, unless the patient has an exceptionally high activity level and health status that is comparable to their peers at a younger age. For patients who are 65–80, hemiarthroplasty remains a viable option but will depend on the surgeon’s evaluation of the patient history, and medical and surgical factors.

The incidence of acetabular wear, factors affecting its occurrence, and subsequent conversion to THA following hip hemiarthroplasty have been variably presented in the literature. Moon et al. [27] found a significantly high conversion rate of hemiarthroplasty to THA in patients younger than 60 years (38.6%). The authors found that younger age at the time of surgery, higher body mass index (BMI), more avascular necrosis of the femoral head, and a smaller prosthetic femoral head contributed to the occurrence of acetabular erosion and subsequent conversion. Macheras [15] reviewed 1410 bipolar HAs at a mean age of 77.2 years and a mean follow-up of 3.2 years and found an incidence of acetabular wear and conversion to THA of 6.13%, 4.22%, and 1.96% for age groups 70–75; 75–80; and more than 80, respectively. The author concluded that ages younger than 75 years were correlated most with the occurrence of wear, while other factors such as sex distribution, injury side, fracture pattern, BMI, ASA score, bipolar head diameter, and leg length discrepancy were not found to be significant in affecting the incidence of erosion and conversion. The author recommended THA for patients younger than seventy-five.

Schivai et al. [16] found a conversion rate of 19 cases in 309 bipolar hemiarthroplasty cases (6.14%), which correlated most with the use of smaller femoral heads of less than 48 mm in diameter, due to the higher polar wear with smaller femoral heads. The authors recommended precise measurement of the native femoral head before choosing the bipolar head and carefully avoiding undersizing the head. Theil et al. [19] found a conversion rate of 5/112 patients (4.6%) who underwent proximal femoral replacement bipolar hemiarthroplasty for patients with malignant bone tumors. The authors found that only ages under 40 and longer follow-ups (63 vs. 43 months) correlated with conversion. Emri et al. [20] found a conversion rate of 36/290 (17.2%) in a series of bipolar HA cases with a mean age of 77.4 years at a mean follow-up of 5 years. The authors found that the use of a prosthetic femoral head 1–2 mm smaller than the native femoral head, an LLD of more than 2 Cm, and a low T score correlated with the occurrence of wear. Grosso et al. [28] found a conversion rate of 5/686 hemiarthroplasty cases (0.7%) at a minimum of a 2-year follow-up and did not find variations with regard to erosion between bipolar versus unipolar cases. Zucchini et al. [29] compared bipolar HA to THA for the reconstruction of proximal femoral tumors using a proximal femoral mega prosthesis. The authors reported a 12.2% incidence of conversion to THA in the patients who received bipolar HA, with a reduced risk of conversion in patients who were younger than 35 or older than 65 and an increased risk of conversion in patients who were affected by a primary bone tumor. Compared to the previous reports, the incidence of acetabular wear and conversions in our study is exceptionally low, which may be attributed to the larger study cohort, the higher mean age of the study population, and the high number of cases that died within the early years following surgery. The effect of femoral head size on symptomatic acetabular erosion did not seem to be significant in our study, given the overall low incidence of erosion in relation to the large sample size, and the mean femoral head size in the symptomatic acetabular erosion cases, with 8/12 cases having femoral head sizes of 48 or more (Table 4). The prosthetic femoral head size depends on the native femoral head diameter and utilizing a prosthetic femoral head of a size smaller than the native size may be more important than the size itself.

While there is a theoretical increase in acetabular wear with unipolar heads compared to bipolar heads [30,31,32], these increased wear rates tend to be comparable in the longer-term follow-up [33] and may not be necessarily translated into better outcomes or decreased revisions [28,34,35,36].

Since the current study reports a relatively small number and incidence of symptomatic erosion and conversions, we could not draw significant statistical conclusions regarding the potential risk factors that may lead to accelerated acetabular wear. The most interesting finding is, however, the short time until presentation with pain and acetabular wear in most of the cases, as symptomatic presentation mostly occurred within the first 1–3 years in the cases who had this complication. It is well understood that acetabular wear and arthritic changes would evolve as the patient continues to live and function actively after surgery, but the finding of early presentation in patients who had this complication raises concerns about the condition of the acetabular cartilage before surgery. Although this was not particularly assessed in our study, the presence of damaged acetabular cartilage at the time of surgery may represent a potential risk factor that could lead to accelerated acetabular wear after hemiarthroplasty. Surgeons may ideally be prepared to shift to THA when performing hemiarthroplasty if the acetabular cartilage is found to be questionable or found to have a macroscopic injury.

Considering our results, given the patient’s medical history and life expectancy, and the reported high mortality rate after hip fractures, hemiarthroplasty remains perhaps the ideal treatment option for femoral neck fractures in elderly, less active patients over 65. For patients younger than 65, hip hemiarthroplasty is a rare treatment option and its use in such patients depends on several factors such as patients’ activity level, medical comorbidities, cognitive function, and life expectancy. The surgeon must weigh the treatment choice considering the patient’s medical and socio-economic factors.

Limitations of the study include its retrospective nature, the wide variations in implant systems, the large number of physicians and subsequent variation in the surgical approaches and methods, and the relatively small number of conversions which could not allow for significant subgroup analyses for the evaluation of obvious risk factors for wear. The inconsistent follow-up durations among all patients and the relatively short mean follow-up in all the study cohorts, which is attributed to the fact that more than one-third of the cases in this study died within three years of surgery, could have potentially affected the outcomes in this study. The wide study duration (from 1988 to 2023) may have affected the proper indications of HA in this study. Also, we did not assess the return to activities postoperatively nor did we compare it to the patient’s pre-injury activity level, considering the patients’ age and the expected reduction in activity levels after hip fractures, which may have potentially influenced the study outcomes. However, the relatively large population size and restriction to a single health system may contribute significant evidence regarding the incidence of acetabular erosion and conversion in hip hemiarthroplasty cases.

## 6. Conclusions

Hip erosion and subsequent conversion to THA do occur in a relatively small number of patients older than 65 years of age who are candidates for hip hemiarthroplasty. As most of the cases of symptomatic acetabular wear in this study presented early after surgery, the question regarding the condition of acetabular cartilage at the time of surgery may be an important factor that contributes to the development of early acetabular wear. In patients who are younger than 65, hip hemiarthroplasty is a rare solution and its use at such a young age depends on several conditional factors.

## Figures and Tables

**Table 1:** Age categories in the study population per total cases.

Age at the Time of Surgery	Less than 65	Patients 65–80	Patients Older than 80
Number (cases)	172	752	1553
Percentage	6.9%	30.3%	62.7%
Mean age	57.6	74.2	87.7
Mean follow-up	4.9 years	3.85 years	3.5 years
Incidence of symptomatic acetabular erosion	3/172 (1.7%)	5/752 (0.66%)	4/1553 (0.26%)
	Chi-square test: *p* = 0.0199 (statistically insignificant)		

**Table 2:** Mortality in the study cases.

Mortality at	Number of Cases	Percent
3 months	327	13.9%
1 year	558	22.5%
3 years	884	35.7%
5 years	1054	42.5%

**Table 3:** Follow-up terms per study population.

Follow-Up Term [21]	Short Term: Less than 2 Years	Midterm 2–10 Years	Long Term: Longer than 10 Years
Number of cases	973	1350	154
Average follow-up (Y)	0.8 Y	4.7 Y	12.9 Y

**Table 4:** Characteristics of patients who complained of chronic pain and acetabular erosion (excluding cases with dislocation, infection, or fractures).

Pt	Sex	Age (Y)	BMI	Implant Type	Symptomatic Acetabular Erosion per Implant Type & Statistical Comparison	Femoral Head Size	Stem Collared(C) /Uncollared (U)	Time Until Presentation (Pain) (M)	Medical Comorbidities	Management	Follow-Up (M) from the Index HA Surgery
1	F	83.7	25	UNI	5/1109 (0.45%)	46	C	11.54		Conservative	25.4 M
2	F	80.2	26	UNI		50	C	4.17	Hypothyroid, Dementia, MS	THA	Died after 79.3 M
3	F	83.3	18.08	UNI		44	C	1.48	Hypothyroid	Conservative	69
4	F	79.78	26	UNI		48	U	35.4		Conservative	47.5
5	F	66.89	26.54	UNI		48	U	2.76		Conservative	43.6
6	F	64.7	19.54	BIP	7/1368 (0.5%) Chi-square test: *p* = 0.8 (not statistically significant)	48	C	4.3	Osteoporosis	THA	146.25
7	F	61.78	24.11	BIP		53	C	146.48	Osteoporosis, Hypothyroid	Conservative	Died after 150 M
8	F	66.19	23.81	BIP		44	C	18.19	Heavy Smoking, COPD	THA	Died after 31.9 M
9	M	47.1	22.46	BIP		40	C	61.6	Spastic CP	Conservative	177 M
10	M	76.58	27	BIP		54	C	2.5	COPD, Epilepsy	THA	Died after 143.88 M
11	M	66.4	28	BIP		50	C	15.36		THA	215 M
12	M	91.99	26.71	BIP		54	C	3.22		Conservative	Died after 6 M

## Data Availability

Data are available with the authors and can be provided upon a reasonable request after fulfilling an institutional data use agreement.
